# A Distinct Boundary between the Higher Brain’s Susceptibility to Ischemia and the Lower Brain’s Resistance 

**DOI:** 10.1371/journal.pone.0079589

**Published:** 2013-11-06

**Authors:** C. Devin Brisson, Mark K. Lukewich, R. David Andrew

**Affiliations:** Department of Biomedical and Molecular Sciences, Queen’s University, Kingston, Ontario, Canada; National University of Singapore, Singapore

## Abstract

Higher brain regions are more susceptible to global ischemia than the brainstem, but is there a gradual increase in vulnerability in the caudal-rostral direction or is there a discrete boundary? We examined the interface between `higher` thalamus and the hypothalamus the using live brain slices where variation in blood flow is not a factor. Whole-cell current clamp recording of 18 thalamic neurons in response to 10 min O_2_/glucose deprivation (OGD) revealed a rapid anoxic depolarization (AD) from which thalamic neurons do not recover. Newly acquired neurons could not be patched following AD, confirming significant regional thalamic injury. Coinciding with AD, light transmittance (LT) imaging during whole-cell recording showed an elevated LT front that initiated in midline thalamus and that propagated into adjacent hypothalamus. However, hypothalamic neurons patched in paraventricular nucleus (PVN, n= 8 magnocellular and 12 parvocellular neurons) and suprachiasmatic nucleus (SCN, n= 18) only slowly depolarized as AD passed through these regions. And with return to control aCSF, hypothalamic neurons repolarized and recovered their input resistance and action potential amplitude. Moreover, newly acquired hypothalamic neurons could be readily patched following exposure to OGD, with resting parameters similar to neurons not previously exposed to OGD. Thalamic susceptibility and hypothalamic resilience were also observed following ouabain exposure which blocks the Na^+^/K^+^ pump, evoking depolarization similar to OGD in all neuronal types tested. Finally, brief exposure to elevated [K^+^]_o_ caused spreading depression (SD, a milder, AD-like event) only in thalamic neurons so SD generation is regionally correlated with strong AD. Therefore the thalamus-hypothalamus interface represents a discrete boundary where neuronal vulnerability to ischemia is high in thalamus (like more rostral neocortex, striatum, hippocampus). In contrast hypothalamic neurons are comparatively resistant, generating weaker and recoverable anoxic depolarization similar to brainstem neurons, possibly the result of a Na/K pump that better functions during ischemia.

## Introduction

There is a well recognized but poorly understood caudal-to-rostral increase in the brain`s vulnerability to neuronal injury caused by metabolic stress [1][2][3] [4]. Global brain ischemia caused by heart attack or near-drowning can leave a functional brainstem while `higher` regions are severely compromised [4], leading to the persistent vegetative state (PVS). Maintained brainstem function with minimal higher brain activity in PVS patients is confirmed by case studies of global ischemia using MR imaging [5][6][7] as well as numerous studies measuring regional metabolism [8]. 

In response to global ischemia, thalamic neurons in rat [9] [10] and dog [11] are injured as are other `higher` neurons in neocortex, hippocampus and striatum. Despite similarly reduced blood flow in the dog, brainstem neurons show comparatively little damage [11]. How does the brainstem survive? Unlike higher brain regions such as thalamus, the adult rat brainstem does not support strong spreading depolarizations [12] unless chemically depolarized [13]. Such events promote acute neuronal injury in stroke and head trauma[14]. In support, we recently showed that a population of neurons in the supraoptic nucleus (SON) of the hypothalamus resists acute injury caused by O_2_/glucose deprivation (OGD) compared to vulnerable neocortical pyramidal neurons [15]. We proposed that this was because the hypothalamus, like brainstem [16] [15], supports only a weak version of the propagating (and damaging) anoxic depolarization (AD) recorded in neocortex, striatum, hippocampus, thalamus and cerebellar cortex. 

Bureš and Burešova [17] observed abrupt rises in extracellular potassium [K^+^_o_] in midline thalamus following terminal anoxia in the rat, but only a small rise in adjacent hypothalamus. In response to Na^+^/K^+^ pump inhibitors in thalamus, intracellular recordings demonstrate a sudden and large inward current representing anoxic depolarization (AD)[18] but only a gradual depolarization in neurons of the hypothalamic SON [15]. Also, exposure of thalamic neurons to elevated [K^+^_o_] caused a prominent and propagating wave of spreading depression (SD) in midline thalamus but only a small signal in lateral hypothalamus [19]. Therefore there may be a distinct border where spreading depolarizations, which promote ischemic damage in higher brain, are only weakly generated in the lower brain.

 Here we examine this possibility using whole-cell patch recording from single neurons combined with light transmittance imaging. The use of brain slices rules out regional differences in blood flow that may affect neuronal survival. AD propagates across neocortex, hippocampus and striatum, leaving swollen cell bodies and beaded dendrites in its wake both in brain slices[20] and whole isolated cortical preparations [21]. In support [22], have shown in vivo that dendritic beading correlates with a dramatic wave of increased light reflectance (beading scatters light) imaged near the surface of intact mouse neocortex following brief global ischemia. It is the initial swelling of neurons and astrocytes during OGD and subsequent dendritic beading [23] that underlies the LT imaging utilized here to compare AD onset and propagation in thalamus and hypothalamus.

## Materials and Methods

### Brain Slice Preparation

Sprague-Dawley rats (male, age 3-10 weeks; Charles River, St.-Constant, PQ) were housed and sacrificed under a protocol approved by the Queen`s University Animal Care Committee. Rats were decapitated by guillotine. Following craniotomy, the brain was quickly removed and immersed in ice-cold and oxygenated (95 % O_2_, 5 % CO_2_) artificial cerebral spinal fluid (aCSF) composed of (in mM) 240 sucrose, 3.3 KCl, 26 NaHCO_3_, 1.3 MgSO_4_.7H_2_O, 1.23 NaH_2_PO_4_, 11 D-glucose and 1.8 CaCl_2_. Using a Leica 1000-T vibratome, 400 µm slices were cut in the sucrose aCSF through the coronal plane and then incubated in regular aCSF (equimolar NaCl replacing sucrose above) at 35°C for at least 1 hour. Slices were then transferred to a recording/imaging chamber where they were submerged in flowing aCSF (3 ml/min) at 36°C ± 0.5°C. The aCSF osmolality was 295 mOsm at pH 7.4. 

### Electrophysiology

Visually guided whole-cell patch recordings were obtained using micropipettes pulled from borosilicate glass (outside diameter 1.2 mm, inside diameter 0.68 mm; World Precision Instruments) to a resistance of 3-6 MΩ. The internal pipette solution contained (in mM) 125 K- gluconate, 10 KCl, 2 MgCl_2_, 5.5 EGTA, 10 HEPES, 2 Na-ATP and 0.1 CaCl_2_ (pH was adjusted to 7.3 with KOH). A 14 mV junction potential was corrected prior to achieving whole-cell configuration. All recordings were acquired in current clamp mode of an Axoclamp 2A amplifier and a Digidata 1322 A/D converter (Axon instruments). Clampex 10 software (Axon instruments) was used for data acquisition with subsequent analysis using Clampfit 10 software. Sampling frequency was 10 kHz and low pass filtering was with an external Bessel filter (LPF 202a; Axon Instruments) at 2 kHz. After obtaining whole-cell recordings, slices were simultaneously imaged (below) while exposed to oxygen glucose deprivation (OGD). The OGD aCSF was of similar composition to control aCSF, except for substituting of 95 % O_2_/5 % CO_2_ bubbling of aCSF with 95 % N_2_/5 % CO_2_. In addition, 11 mM glucose was reduced either to 0 mM or 1 mM glucose with osmotic adjustment using NaCl. Occasionally, neurons in hypothalamus and brainstem were exposed to multiple applications of OGD and/or newly acquired recordings obtained post-OGD in the same slice. ACSF for inducing spreading depression (SD) contained 9.6, 26 or 52 mM KCl replacing equimolar NaCl. All 3 treatments short-circuit the Na/K pump, depolarizing a healthy resting potential. OGD deprives the pump of fuel (ATP), ouabain binds to the pump itself, and high K^+^ depolarizes the neurons (as shown by the Nernst equation) thereby bypassing the pump.

### Imaging changes in light transmittance (∆LT)

We imaged ΔLT in live coronal brain slices during 10 minutes of OGD. A front of *cell swelling* is imaged as an increase in LT during AD initiation and propagation in brain slices. *Dendritic damage* is imaged as decreased LT in the wake of the AD front. This is caused by dendritic bead formation which scatters light and has been confirmed in numerous papers ([24] [25] [20]. Neurons were visualized using near-infrared illumination and Dodt gradient contrast optics (Luigs and Neumann, Ratingen, Germany) through an upright microscope (Axoscope 2FS, Zeiss) with a 40x immersion objective lens. Video images were captured with a cooled charge-coupled device (Hamamatsu C4742) using Imaging Workbench 6 software (Indec Biosystems Inc.). Each image of a video series consisted of 16 averaged frames acquired at 20 Hz. The first image of the series was the control transmittance (*T*_*cont*_) which was subtracted from each of the subsequent images (*T*_*exp*_) in the series. The difference signal was normalized by dividing by *T*_*cont*_, which varies across the slice depending on the zone sampled. For example, *T*_*cont*_ was lower in white matter than gray matter. This value was then presented as a percentage of the digital intensity of the control image of that series. That is, ∆LT = [(*T*_*exp*_ - *T*_*cont*_)/ *T*_*cont*_ ] x 100 = [∆T/T] %. The change in LT was displayed using a pseudocolor intensity scale. The slice image in bright field was displayed using a gray intensity scale. 

### Statistical Analysis

Neuronal parameters were analyzed in detail if the recording displayed stable resting membrane potentials and if the series resistance could be sufficiently compensated. All were data presented as means +/- standard deviation.

## Results

### Thalamic Neurons

**Figure 1:**
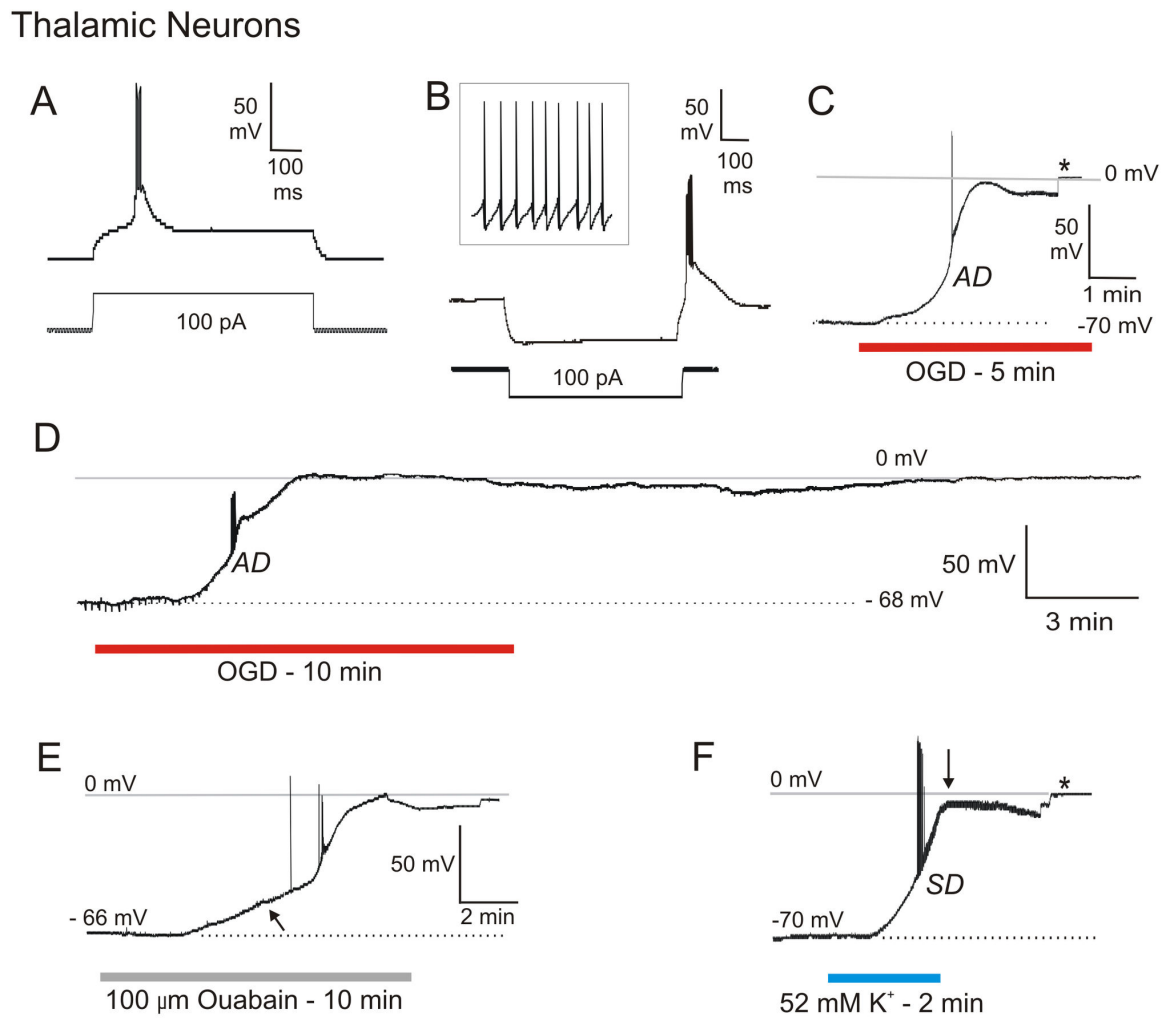
Thalamic neurons undergo terminal anoxic depolarization (AD) or spreading depression (SD). A) A thalamic neuron held hyperpolarized by 10 mV characteristically fires a brief burst when step depolarized. B) A hyperpolarizing current pulse evokes a burst of firing but when depolarized from positive resting potentials, the same neuron fires tonically (inset). C) In response to 10 minutes of OGD, the neuron gradually depolarizes before rapid onset of AD to near-zero millivolts but the recording was then lost (asterisk). D) Another thalamic neuron during 10 minutes of OGD undergoes rapid AD to near-zero millivolts. Following return of control aCSF the cell could not repolarize. At the same time the input resistance of the cell is near zero megaohms. Higher neurons do not recover from 10 minutes OGD in slices at 35-36^O^C. E) Response 100 µM ouabain exposure for 10 minutes. The neuron slowly depolarizes (arrow) followed by a rapid AD-like event to near-zero millivolts. Following return to control aCSF the cell did not recover. F) In response to elevated [K^+^]_o_ exposure, the neuron rapidly depolarizes before reaching a brief plateau as action potentials inactivate. The rapid depolarization of spreading depression (SD) drives the neuron to near-zero millivolts (arrow). Control aCSF is returned to the slice and the neuron then begins to repolarize before it is lost (asterisk). All thalamic recordings are from the anteromedial ventral (AMV) nucleus except C, which is from the interanteromedial (IAM) nucleus.

Eleven neurons in anteromedial ventral (AMV) nucleus were monitored by whole-cell current clamp during simulated stroke (Figure 1; Table S1). The patch pipette was visually placed within AMV and the cells targeted based on their elongated shape and length of 20 to 30 µm. When steadily hyperpolarized below -65 mV, a depolarizing current step evoked a low threshold potential driving a burst of action potentials, (Figure 1A). The response was also evoked with a hyperpolarizing pulse (Figure 1B). When depolarized above -55 mV, these cells fired tonically (Figure 1B, inset). The mean resting potential of the 11 AMV cells was -70 ± 3.2 mV. Mean input resistance and action potential amplitude were 81 ± 20 MΩ and 78 ± 5.9 mV, respectively. Exposing these 11 neurons to OGD elicited an abrupt anoxic depolarization (AD) with a mean slope of 2.0 ± 0.8 mV/s and a mean onset time of 217 ± 26 seconds (Figure 1C; Table S1). The average maximal depolarization reached during AD, was -4 ± 2.5 mV. Average resting membrane potential following OGD could not be determined, as none of the whole-cell recordings from the AMV nucleus could be maintained (asterisk, Figure 1C). 

To show that AMV neurons were typical thalamic cells, additional recordings were made in the lateral (n = 3), interoanteromedial (n =3), anteromedial (n = 1) and submedial (n = 1) thalamic nuclei. Each of these neurons showed abrupt AD in response to OGD (Figure 1D, Table S2). This neuron from the IAM nucleus showed no recovery following 10 minutes OGD. The mean onset time of this second group of thalamic neurons was 243 ± 31 seconds with a mean slope of 6.6 ± 5.1 mV/s. Mean resting potential, maximum depolarization, action potential amplitude and input resistance values were similar to thalamic neurons from the AMV nucleus (Table S2). Even after an hour of normal aCSF exposure post-AD, it was impossible to obtain new thalamic recordings from the electrically silent thalamus.

### Light Transmittance (LT) Imaging of Thalamus during OGD

**Figure 2:**
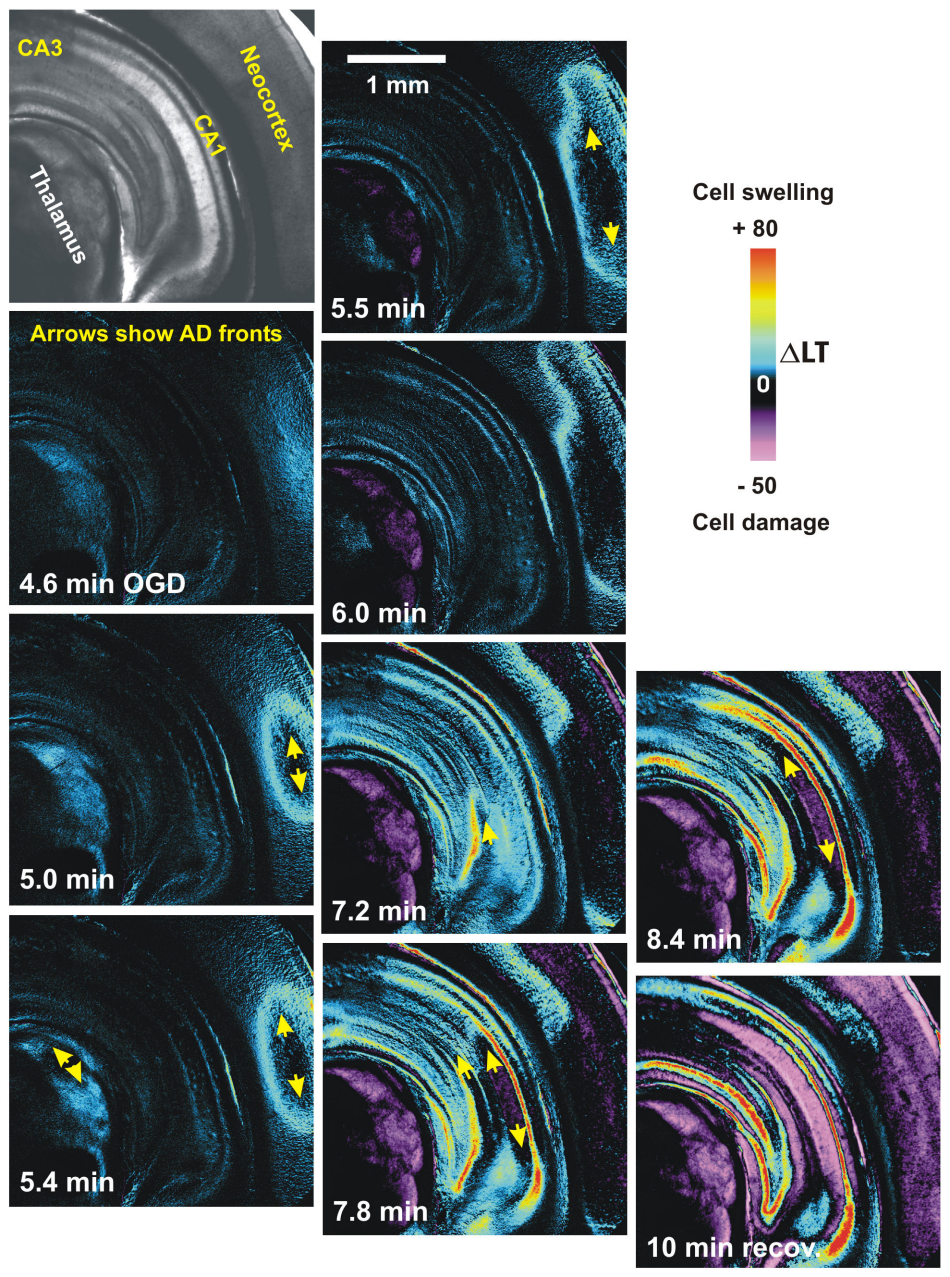
Light transmittance (LT) imaging of AD propagating through higher brain regions. Coronal brain slice from adult rat exposed to OGD for 10 min at 34°C. Strong and damaging AD is generated in the thalamus. AD initiates in thalamus (5.4 minutes) as well as neocortex and the dentate and CA1 regions of hippocampus. Cell swelling, associated with AD onset and propagation (arrows), evolves to acute cell damage which scatters light (purple) in the wake of AD over several minutes.

**Figure 3:**
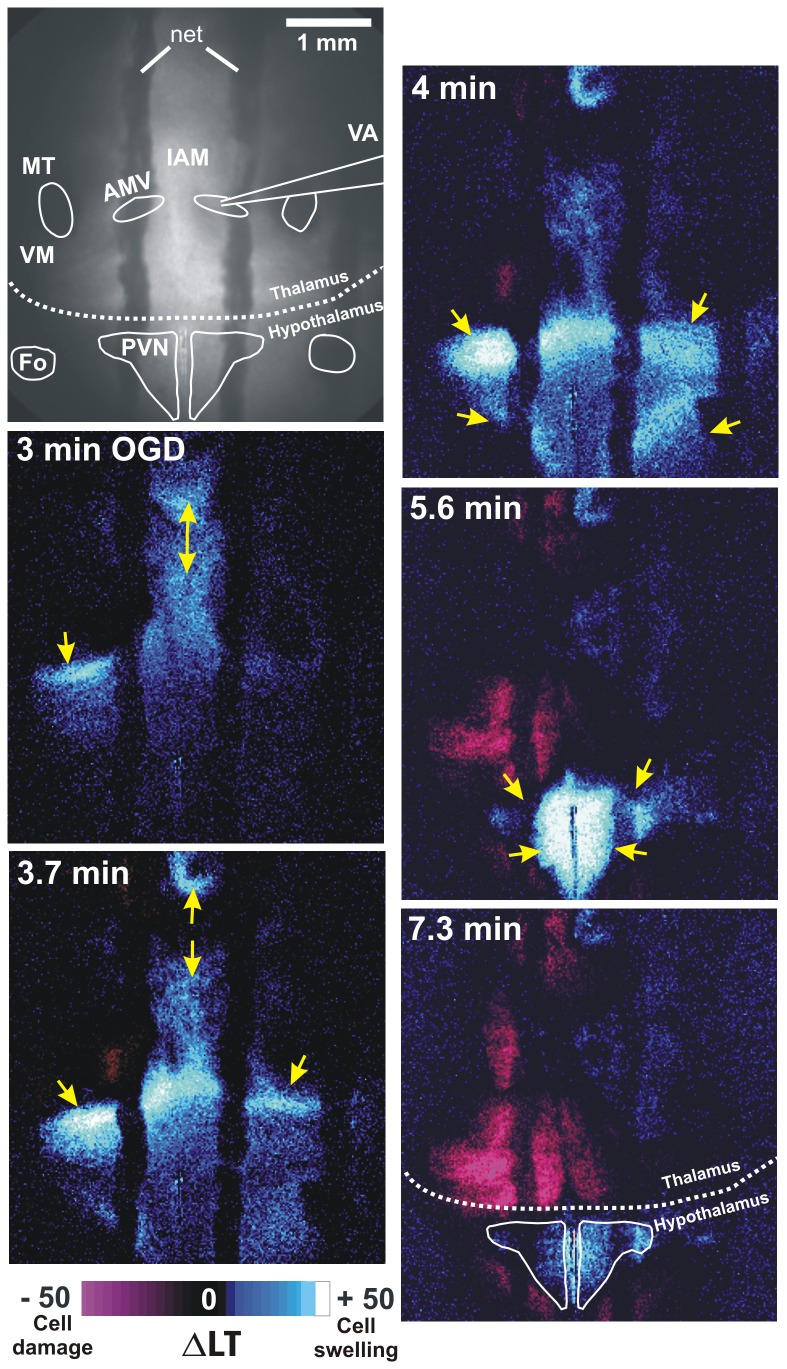
LT imaging of AD propagating through thalamus and hypothalamus. A) The AD front initiates medially and laterally (arrows) below the mammillo-thalamic tract (MT) at 3 minutes of OGD, before propagating dorsally and ventrally (arrows). At 4 minutes the ventral wave is joined by a weak wave from lateral hypothalamus that simultaneously engages PVN. At 5.6 minutes both PVNs swell (blue-white) which dissipates as thalamic injury develops (purple). AMV, anteromedial ventral thalamic nucleus; IAM, interoanteromedial nucleus; VA, ventroanterior nucleus; VM, ventromedial nucleus; F, fornix.

Imaging of coronal slices that included neocortex, striatum, hippocampus and thalamus revealed robust front of AD coursing through these gray regions within 4-7 minutes of OGD (Figure 2). In the wake of AD all of these higher regions including thalamus display reduced LT indicating neuronal damage. Simultaneous LT imaging of the thalamic field during whole-cell recording demonstrated a sudden increase in ∆LT that usually initiated medially near the IAM nucleus, before spreading in dorsal and ventral directions (Figure 3). The propagating wave of elevated LT engaged the AMV at ~ 3 minutes, which coincided with the fast component of the AD in the whole-cell recording (Figure 1C, D). After propagating through the thalamus and adjacent lateral hypothalamic areas, the AD front converged upon and engaged the paraventricular nucleus (PVN) in hypothalamus (Figure 3, at 4 min and 5.6 min). Within the PVN a robust and sustained increase in LT was measured during OGD, a consistent observation in our slices. Following return to control aCSF, the PVN signal returned to near-baseline indicating recovery (Figure 3 at 7.3 min) In contrast, adjacent thalamus displayed reduced LT indicating dendritic damage (purple in Figure 3 at 7.3 min). 

Exposing the slice to 100 µM ouabain for 10 minutes elicited an abrupt AD-like response by thalamic neurons to near zero millivolts, similar to OGD (Figure 1E). The initial slow phase of the depolarization (arrow) led to a faster phase. Again as the slice swelled, the thalamic cell recording could not be maintained (asterisk, Figure 1E).

Exposing thalamic slices to 52 mM [K^+^]_o_ for ~2 minutes elicited an abrupt depolarization of the thalamic neuron to near zero millivolts (Figure 1F), which was diagnostic of SD initiation. Upon return of control aCSF (arrow), membrane potential began to repolarize but again the recording could not be maintained (asterisk, Figure 1F). We found thalamic recordings to be the most difficult CNS neurons to hold once a spreading depolarization was underway.

### Hypothalamic Neurons

**Figure 4:**
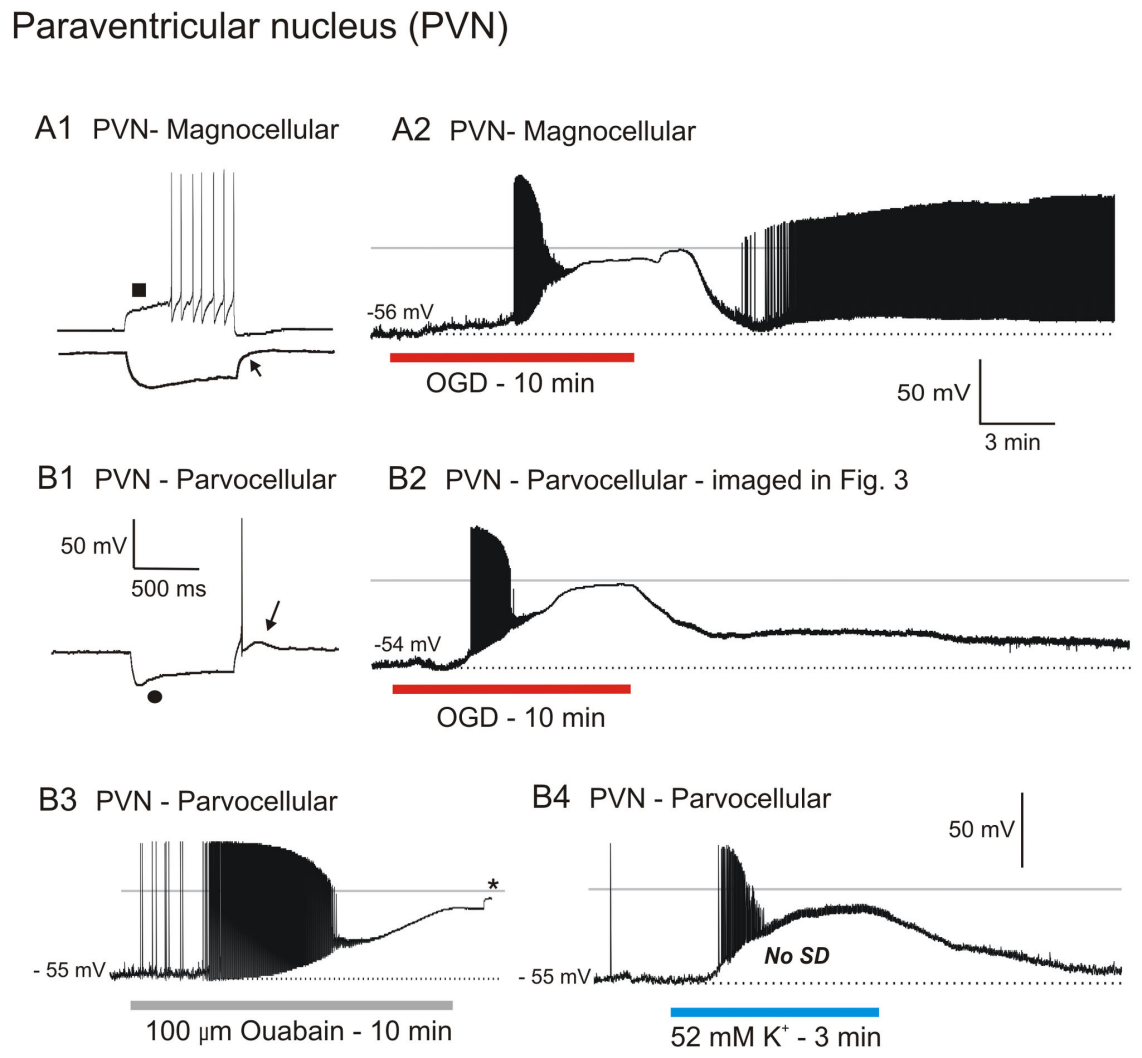
Neurons of PVN resist depolarization evoked by OGD, ouabain or elevated [K^+^]_o_ exposure and subsequently recover. A1) MNCs display a characteristic delay in firing (arrowhead) when depolarized from negative resting potentials and a delay in return to baseline (arrow) in response to a hyperpolarizing current step. A2) During 10 minutes of OGD, an MNC gradually depolarizes before reaching a plateau as action potentials inactivate. Following return of control aCSF, the MNC rapidly repolarizes with spontaneous action potential firing. B1) A non-bursting parvocellular neuron with characteristic post-inhibitory rebound spike and low-threshold spike (LTS). B2) A parvocellular neuron responds to 10 minutes of OGD by gradually depolarizing before reaching a plateau as action potentials inactivate. The neuron resists further depolarization before slowly approaching zero millivolts. Following return to control aCSF, the neuron repolarizes to near-baseline. B3) A parvo during 10 minutes of ouabain exposure slowly depolarizes as action potentials inactivate. The neuron plateaus and then depolarizes to near-zero millivolts. Following return of control aCSF the recording is lost (asterisk). B4) A parvo in response to 3 minutes of 52 mM [K^+^]_o_ exposure depolarizes before reaching a plateau as action potentials inactivate. No SD is generated. Following return of control aCSF it repolarizes to near its original resting potential.

 Magnocellular Neuroendocrine Cells (MNCs) in Paraventribular Nucleus (PVN). Eight MNCs were monitored by whole-cell patch during simulated stroke. The patch pipette was visually placed within the magnocellular region of the PVN, and the cells identified based on their ellipsoid shape and large diameter of 20 to 30 µm. The mean resting membrane potential of the 8 cells was -51 ± 3.9 mV (Table S3). Input resistance and action potential amplitude were 517 ± 173 MΩ and 86 ± 4.9 mV respectively, similar to other studies[26]. All MNCs displayed characteristic frequency-dependent action potential broadening, linear IV curves (not shown) and transient A-type outward K^+^ current when depolarized from holding potentials more negative than -70 mV (Figure 4A, square) [27]. A characteristic delay in return to baseline which followed a hyperpolarizing current pulse (Figure 4A1, arrow) was also diagnostic of MNCs. 

Ten minutes of OGD elicited a gradual depolarization of MNCs, reaching a mean plateau of -23 ± 6.0 mV after firing ceased, presumably due to Na^+^ channel inactivation (Figure 4A2). Upon reaching this plateau, MNCs resisted further depolarization for 168 ± 44 seconds, before maximally depolarizing to -9 ± 6.5 mV (Table S3). Post-OGD, 4 recordings that were held returned to -42 ± 2.9 mV, representing an 84 % recovery of membrane potential. Mean input resistance and action potential amplitude post-OGD were 458 ± 112 MΩ (89 % recovery) and 71 ± 4.5 mV (82 % recovery) respectively.

Newly acquired MNCs could readily be obtained following 10 min of OGD exposure (Table S3). The average membrane potential of three of these was - 52 ± 2.1 mV (100 % control). Input resistance and action potential amplitude were 674 ± 102 MΩ (100 % control) and 88 ± 5.0 mV (100 % control), respectively. These data replicated our previous MNC data from the hypothalamic SON[15]. 

 Parvocellular Neurons in PVN. Twelve parvocellular neurons were monitored during simulated stroke (Figure 4B; Table S4). The patch pipette was visually placed within the parvocellular region of the PVN and small neurons targeted (< 15 µm dia.). Cells were further characterized (Figure 4B1) by inward rectification (circle) and by 1 to 2 rebound spikes (arrow) following a hyperpolarizing current step. Mean resting membrane potential of 12 neurons was -49 ± 4.2 mV (Table S4). Input resistance and action potential amplitude were 704 ± 233 MΩ and 80 ± 8.1 mV, respectively as reported in other studies [26]. 

Exposing parvocellular neurons to 10 minutes of OGD elicited a gradual depolarization to -26 ±4.4 mV. Firing ceased likely due to Na^+^ channel inactivation (Figure 4B2). The neurons then resisted further depolarization for 74 ± 49 seconds, before depolarizing to -6 ± 3.2 mV (Table S4). The average membrane potential of three parvocellular neurons that were maintained following 10 minutes of OGD exposure was -38 ± 6.7 mV (Table S4), representing a 78 % recovery of membrane potential. Input resistance and action potential amplitude were 469 ± 33 MΩ (92 % recovery) and 82 ± 5.7 mV (95 % recovery), respectively.

 In contrast to thalamus but similar to MNCs, newly acquired `parvo` recordings post-OGD could be readily obtained from PVN (Table S4). Mean membrane potential of these 11 neurons following 10 minutes of OGD was -44 ± 5.4 mV, representing 90 % of the resting membrane potential of parvos not previously exposed to OGD (n = 11). Input resistance and action potential amplitude were 603 ± 154 MΩ (86 % control) and 80 ± 7.6 mV (100 % control), respectively. Simultaneous LT imaging of the PVN (Figure 3, 5.6 min) during whole-cell recording of a parvo (Figure 4A2) showed that the elevated LT corresponding with AD onset in the single cell. The mean AD onset in PVN in 6 slices was 298 ± 57 seconds. Within the PVN sustained increase in LT was measured during OGD, accompanied by gradual depolarization of the recorded parvocellular neuron (Figure 4A2). Upon return of control aCSF, increased LT within the PVN slowly returned to baseline, indicating recovery.

 Exposing parvos to 100 µM ouabain for 10 minutes elicited gradual depolarization, similar to OGD (Figure 4B3). As with OGD, the neuron reached a plateau before further depolarizing to -7 mV. Following return of control aCSF the recording was lost (asterisk).

A 3 minute exposure to 52 mM [K^+^]_o_ elicited depolarization and spike inactivation at -27 mV (Figure 4B3). Unlike thalamic neurons but like MNCs, no spreading depression (SD) was generated. Upon return to control aCSF, the cells repolarized to near-baseline. 

**Figure 5:**
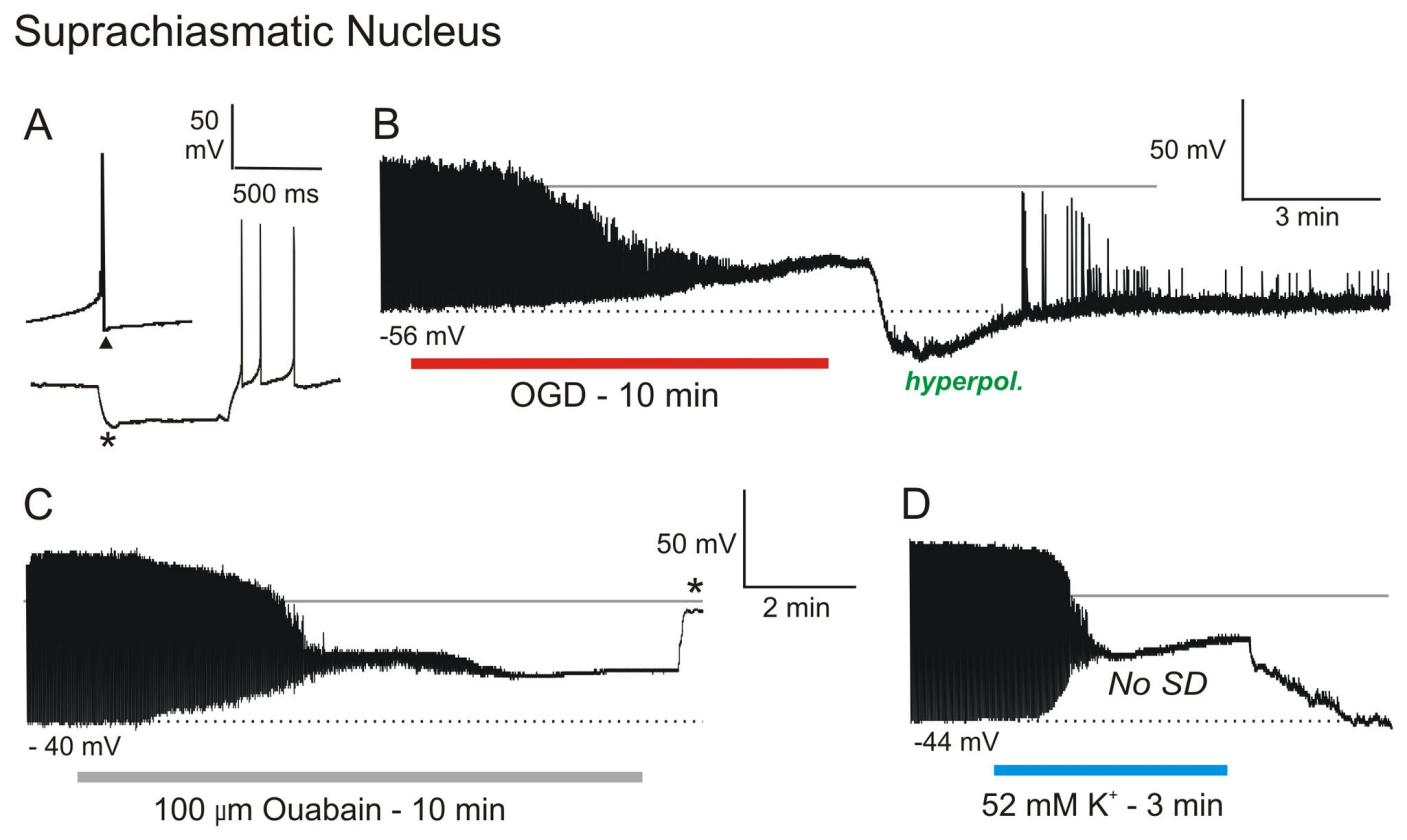
SCN neurons also resist depolarization during OGD, ouabain and 52 mM [K^+^]_o_ exposure and subsequently recover. A) In response to a hyperpolarizing current pulse an SCN neuron shows pronounced inward rectification (arrow) and a biphasic afterhyperpolarizing potential (arrowhead). B) SCN neuron responds to 10 minutes of OGD by slowly depolarizing before reaching a plateau as action potentials inactivate. After return to control aCSF it rapidly repolarizes, followed by a hyperpolarizing undershoot. C) SCN neuron responds to10 minutes 100 µM ouabain similar to OGD but the recording is lost (asterisk) before recovery. D) SCN neuron in responds to 3 minutes of 52 mM [K^+^]_o_. It depolarizes to a plateau as action potentials inactivate, but does not generate SD. Back in control aCSF, the neuron returns to the original resting potential.

 Neurons in Suprachiasmatic Nucleus (SCN). Eighteen neurons from SCN were recorded during simulated stroke (Figure 5; Table S5). The patch pipette was visually placed within SCN and neurons targeted based on their small diameter of < 15 µm. Neurons were further categorized as either type I, II or III based on electrophysiological properties [28]. Type I neurons (n =10) displayed irregular firing frequency and a monophasic afterhyperpolarization (AHP; not shown). Type II neurons (n = 7) displayed regular firing and biphasic AHP’s (Figure 5A, arrowhead). Type III neurons (n = 1) also displayed a burst of action potentials overriding a rebound depolarization following a hyperpolarizing current step (Figure 5A). All 18 SCN neurons showed pronounced inward rectification (asterisk, Figure 5A) with a mean resting potential of -45 ± 4.1 mV (Table S5). Mean input resistance and action potential amplitude were 799 ± 293 MΩ and 76 ± 10 mV respectively, similar to previous studies [29] [28]. 

Exposing SCN neurons to 10 (n = 8) or 15 (n = 10) minutes of OGD, elicited a gradual depolarization, reaching a plateau of -27 ± 2.2 mV as action potential firing ceased (Figure 5B). SCN neurons then resisted further depolarization for 322 ± 187 seconds, before depolarizing to -19 ± 6.9 mV (Table S5). Upon return to control aCSF, SCN cells rapidly repolarized often displaying a hyperpolarizing undershoot (Figure 5B) before returning to baseline potential. The mean membrane potential of the 13 SCN neurons following 10 or 15 minutes of OGD was -51 ± 10.9 mV, an 81 % recovery of membrane potential (Table S5). Input resistance and action potential amplitude were 855 ± 275 MΩ (100% recovery) and 46 ± 12 mV (65 % recovery).

As with other hypothalamic neurons, newly acquired and healthy SCN neurons could be readily patched following recovery from OGD (Table S5). The average membrane potential of 15 new SCN neurons was -57 ± 3.3 mV (97% of control SCN neurons). Input resistance and action potential amplitude were 900 ± 523 MΩ (100% control) and 71 ± 9.0 mV (93 % control).

**Figure 6:**
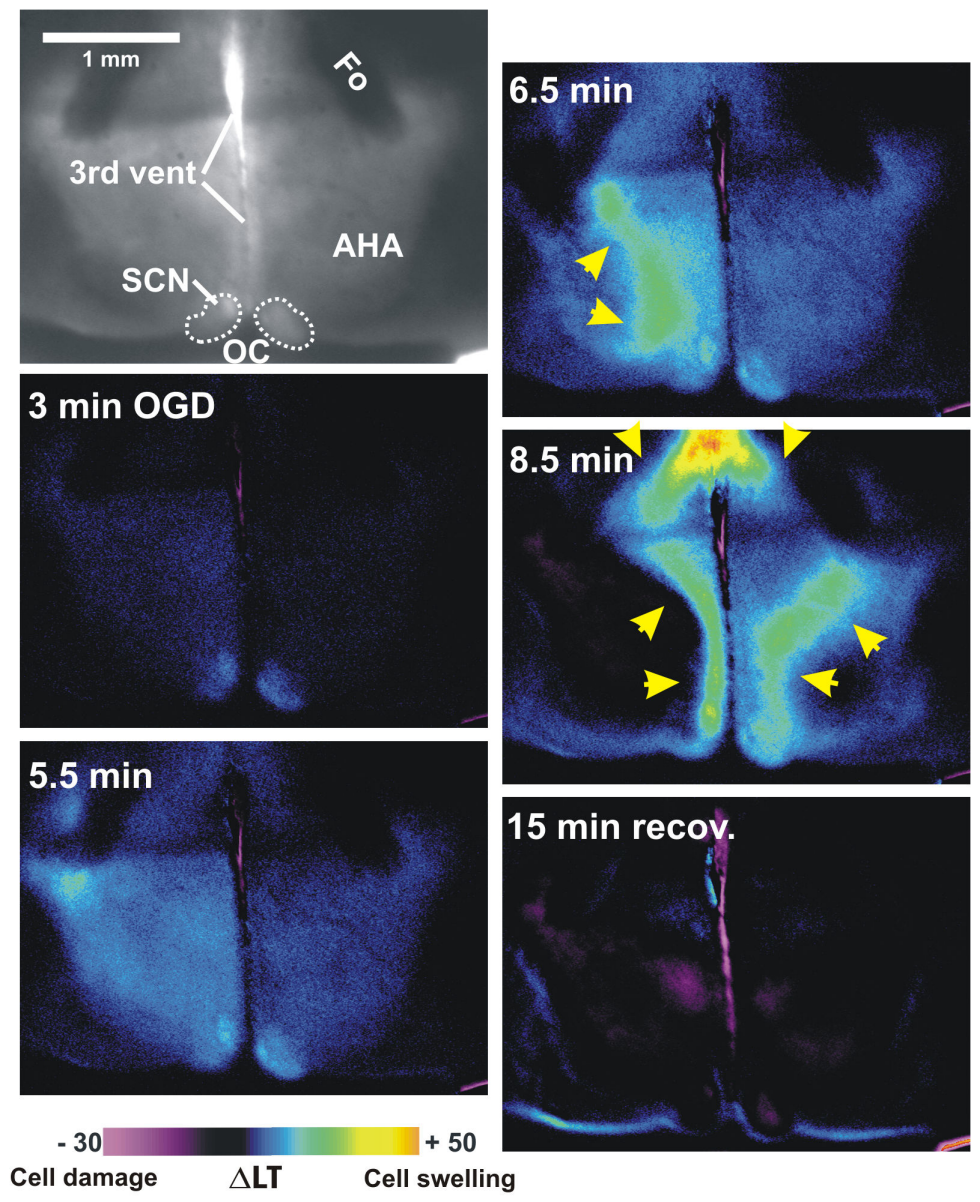
LT imaging of AD propagating through rostral hypothalamus. There is initial cell swelling particularly in SCN and then an AD wave front propagates latero-medially at 6.5 min of OGD (arrows). By 8.5 min, an AD front originating in thalamus invades dorsally (downward arrows). Following 15 min post-OGD, the hypothalamus shows little damage (purple) compared to the higher regions in Figures 3 and 4. AHA, anterior hypothalamic area.

Imaging of the anterior hypothalamus demonstrated a slow LT increase in SCN during 3-6 minutes of OGD (Figure 6). Then an LT front that originated laterally and a second front moving ventrally from medial thalamus propagated through SCN at 431 ± 63 seconds (n = 7 slices). Despite this, return to control aCSF evolved to only minimal LT reductions in the hypothalamus, indicating recovery (Figure 6, 15 min).

Exposing SCN neurons to 100 µM ouabain for 10 minutes elicited a gradual depolarization, similar to OGD (Figure 5C). As with OGD, the neuron resisted depolarization after reaching a plateau, finally depolarizing to approx. - 20 mV. Upon return of control aCSF, the SCN neuron in Figure 5C was lost (asterisk). No hyperpolarization immediately following OGD was observed when ouabain was removed suggesting a fully functional pump is required.

Brief exposure (3 minutes) of SCN neurons to 52 mM [K^+^]_o_ elicited depolarization and cessation of firing before reaching a plateau of -32 mV (Figure 5D). As with the other hypothalamic neuron, no SD was generated. Upon return of control aCSF, SCN neurons repolarized to its original resting potential level, similar to other hypothalamic neurons.

**Figure 7:**
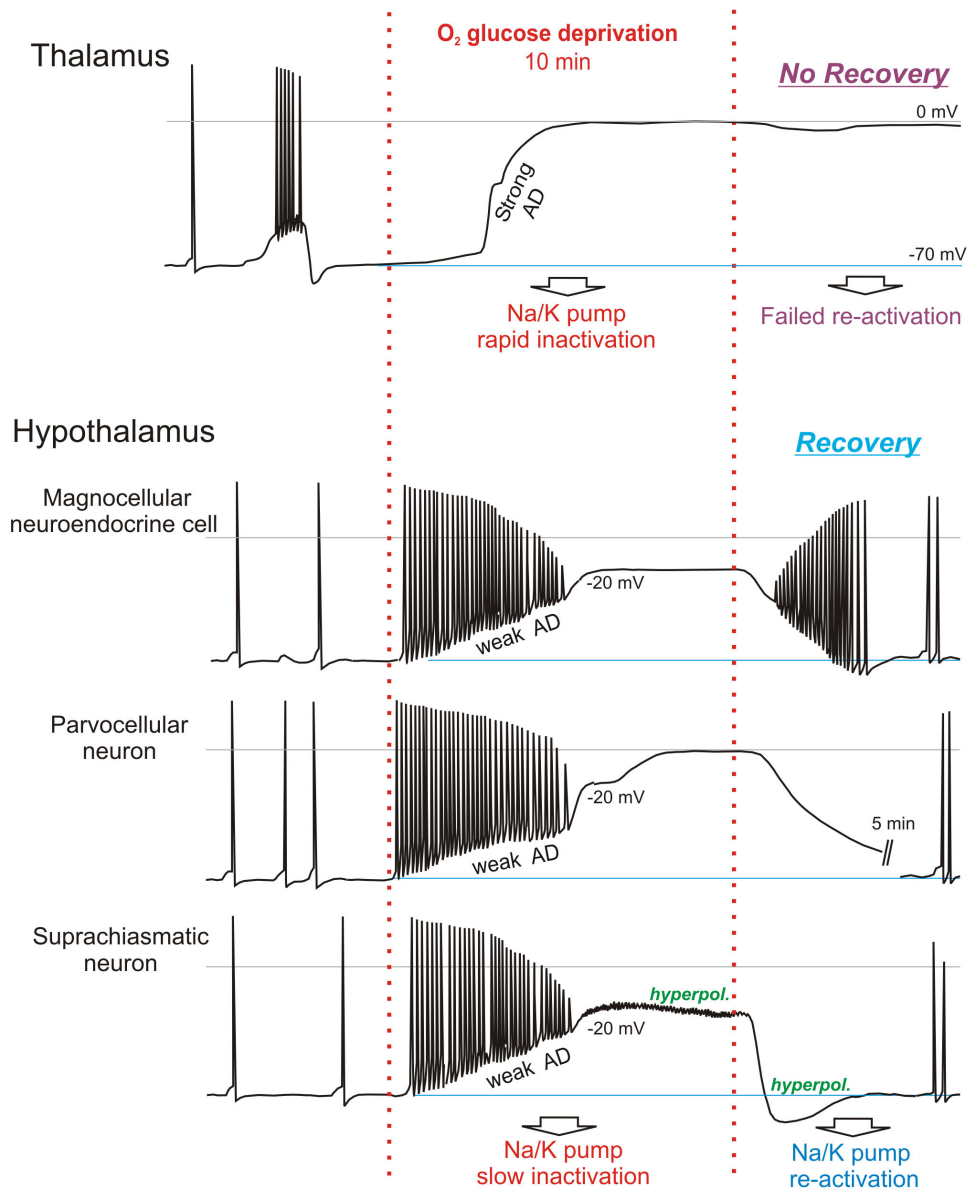
Summary of responses to OGD which varies with neuronal type. Like other neurons recorded in neocortex, striatum, hippocampus and cerebellar cortex, thalamic neurons undergo rapid AD to near-zero millivolts in response to OGD. They do not recover in brain slices exposed to 10 minutes OGD. In contrast, three types of hypothalamic neuron undergo gradual and often incomplete AD that usually plateaus at ~ 20 mV. Moreover these neurons substantially repolarize post-OGD. Some SCN neurons can even hyperpolarize in the face of OGD and this elevated pumping is reflected as a post-OGD hyperpolarization. We propose that hypothalamic neurons possess more resilient versions of the Na^+^/K^+^ pump than thalamic neurons.

Whole-cell data from thalamic (n=18) and hypothalamic neurons (n=38) in response to 10 min OGD are summarized in Table 1. Prior to OGD thalamic neurons displayed more polarized resting potentials and lower cell input resistances. Post-OGD their membrane potentials were near-zero and recordings were usually lost. In contrast, hypothalamic neurons depolarized less and recovered. As well, their AD onset was delayed compared to thalamic neurons. The general response to OGD by each neuronal type in the current study is diagrammed in Figure 7. Note the slow, delayed and recoverable AD displayed by hypothalamic neurons, but not thalamic neurons.

**Table 1:** Whole-cell recording parameters from thalamic (n=18) and hypothalamic neurons (n=38) in response to 10 min OGD.

**Neuron Type**	**Rmp (mV)**	**Rmp Post-OGD (mV)**	**% Rmp Recov.**	**Max Depol. (mV)**	**% AP Ampl. Recov.**	**Rin (MΩ)**	**% Rin Recov.**	**AD Onset (s)**
**Anteromed. Thalamus (n=11)**	-70 ± 3.5	No recovery	N/A	-4 ± 2.5	N/A	81 ± 20	N/A	217 ± 26
**Midline Thalamus (n=7)**	-69 ± 2.9	No recovery	N/A	-3.3 ± 2.4	N/A	141 ± 71	N/A	243 ± 31
**Magnocellular (PVN) (n=8)**	-51 ± 3.9	-42 ± 2.9	84 ± 6.3	-9 ± 6.5	82 ± 11	517 ± 173	89 ± 13	298 ± 57
**Parvocellular (PVN) (n=12)**	-49 ± 4 .2	-38 ± 6.7	78 ± 17	-6 ± 3.2	82 ± 5.8	704 ± 233	92 ± 37	298 ± 57
**SCN neurons (n=18)**	-45 ± 4.1	-37 ± 11	81 ± 22	-19 ± 7	65 ± 18	799 ± 293	100 ± 15	431 ± 63

All neurons were exposed to 10 minutes OGD except 10 of the SCN neurons which underwent 15 minutes OGD. Abbreviations: OGD Dur., oxygen/glucose deprivation duration; [Gluc.], glucose concentration; Rmp, resting membrane potential; Max Depol., maximum depolarization of anoxic depolarization; AP Ampl., action potential amplitude; Rin, whole-cell input resistance. % Rmp recovery was calculated before correcting for a +14 mV junction potential.

## Discussion

### Ischemic vulnerability of the brain decreases rostro-caudally

MR imaging of patients who survive global ischemia but remain in a persistent vegetative state (PVS) , show relatively normal brainstem function but dysfunctional higher brain activity [5] [6] [30] [7], a finding supported by numerous studies measuring regional metabolism [8]. There is also evidence from animal studies that AD which is acutely damaging in higher brain [31] is comparatively weak in brainstem. Specifically, AD arising from respiratory arrest in intact rat brain measured using K^+^-sensitive electrodes [17] showed that the profile of elevated [K^+^]_o_ (representing AD strength) is delayed, is slower to rise, and peaks at lower levels in hypothalamus and brainstem compared to cortex and striatum. As well, global ischemia in dogs for 20 minutes [11] evoked a gradient of neuronal injury assessed 7 days later as follows: neocortex, hippocampus, cerebellum > basal ganglia> thalamus > brainstem. In fact, they detected no injury in midbrain, pons or medulla despite all regions being deprived of blood over the same period. The adult hypothalamus and brainstem do not naturally undergo spreading depression [12] so it is not surprising that they are also less supportive of AD.

Neurons in higher brain undergo strong AD (see Introduction) while hypothalamic and brainstem neurons show weak or no AD [15] [16][32]. This also holds for the less metabolically stressed version of AD, spreading depression (ibid) and in slices is obviously independent of regional variation in blood flow. Specifically, using whole-cell patch and 2-photon microscopy, we have previously reported a clear difference in neuronal responses to OGD between susceptible neocortical pyramidal neurons and more resilient MNCs in the hypothalamic SON [15]. Here we examined if and where a demarcation in susceptibility to AD and SD generation can be identified. We show that the transition from vulnerable to more resilient is at the thalamus-hypothalamus interface. Following 10 minutes of OGD in rodent slices, the thalamus is essentially dead. No new thalamic neurons can be obtained post-OGD, even following up to 1 hour recovery in control aCSF. Thalamic neurons, like those of neocortex [33], CA1 hippocampus [34] [35] [36] and striatum [2] undergo strong and irreversible AD that kills the neurons in brain slices.

In contrast, tens of microns away in hypothalamus we recorded three neuronal types in the paraventricular nucleus (PVN) and suprachiasmatic nucleus (SCN) showing comparative resilience to 10 or 15 minutes of OGD. Theses neurons recover their membrane potential, input resistance and action potential amplitude. Moreover, newly acquired neurons are readily obtained in the hypothalamus following OGD, a feat not possible in adjacent thalamus, even following up to an hour of recovery in control aCSF. Their resting membrane potential, input resistance and action potential amplitude are similar to their neuron counterparts not previously exposed to OGD, further indicating good recovery from OGD. This resilience is similar to that reported in a fourth hypothalamic region, the supraoptic nucleus [15]. That study sampled only one type of `higher` (neocortical) neuron and one type of `lower` hypothalamic (MNC) neuron but there was no way to generalize that hypothalamic neurons resist ischemic resistance. By sampling 3 additional hypothalamic neuronal types and comparing them to nearby thalamic neurons, this study can make that conclusion.

### Imaging LT Changes during OGD

 A robust AD onset measured with current clamp has been consistently correlated with a propagating wave of increased LT passing by the recording electrode [37][38] [15]. During OGD an LT front representing AD onset initiated in the midline thalamus. As AD approached the hypothalamus, it slowed and was usually joined by a separate smaller wave originating in the lateral hypothalamus that converged upon the PVN. These findings are consistent with [K^+^]_o_ measurement of AD strength during terminal anoxia in rat, where a rapid increase (30 - 50 mM) in [K^+^]_o_ was observed in midline thalamus but only a slow, small rise (6 - 10 mM) in lateral hypothalamus [17]. Whole-cell recordings of MNC and parvocellular neurons during OGD showed AD slowly developing as the front converged upon the PVN. The reversible increase in LT observed in PVN during OGD likely involves the swelling of astrocytes that quickly recover post-OGD [23]. MNC somata display no detectable swelling during or after OGD as observed with 2-photon microscopy [15] further confirming minimal damage to MNCs post-OGD. In the SCN during OGD, a slow LT increase likewise coincides with AD onset in the recorded SCN cells. AD onset in SCN (~7 min) was later than in PVN (~5 min), because AD from the ventral hypothalamus invaded PVN earlier than AD from medial thalamus. 

### Spreading Depression (SD) is supported in thalamus but not hypothalamus

Brief exposure of slices to 52 mM [K^+^]_o_, caused rapid depolarization of midline thalamic neurons that quickly approached zero millivolts. Using whole-cell voltage clamp recordings of thalamic neurons in lateral geniculate nucleus [39], demonstrated a sudden large inward current driving SD in response to hypoxia. SD was also induced in thalamus in response to intracerebral injections of KCl [19] or electroconvulsive shock [17]. In both cases SD was stronger in midline thalamus than lateral or posterior regions. Despite a long exposure (3 min) to 52 mM [K^+^]_o_ , in no case did hypothalamic neurons generate SD measured either electrophysiologically or by LT imaging. Although gray matter is continuous between midline thalamus and adjacent hypothalamus, no propagation of normoxic SD was observed into hypothalamus in vivo [17] [19]. Moreover, direct injection of KCl into hypothalamus could not elicit SD, except for a small signal detected in the lateral hypothalamic region . These results are consistent with our findings in supraoptic MNCs of the hypothalamus [15] and brainstem from adult rat [16] showing that these lower regions do not naturally undergo SD but can generate weak AD. 

### Blocking Na^+^/K^+^-ATPase with ouabain mimics OGD in thalamus and hypothalamus

 Exposure to 100 µM ouabain evokes an AD-like response in all neuronal types tested. In thalamus, an initial slow rise in membrane potential was followed by a rapid depolarization to near zero millivolts. Similarly, voltage clamp recordings of thalamic neurons in medial geniculate body (MGB), revealed a sudden large inward current representative of AD, in response to Na^+^/K^+^ ATPase inhibitors strophanthidin or dihydro-ouabain [18]. As during OGD, hypothalamic neurons in the current study only gradually depolarized during ouabain exposure. So as consistently observed in numerous intracellular and imaging studies, for example 38, ouabain exposure re-iterates the OGD response by inducing failure of the Na^+^/K^+^ pump. However, recovery is slower because of the slow washout of ouabain from slices, compared to the rapid re-infusion of oxygen and glucose.

 The striatum supports robust ID even though it is structurally similar to brainstem gray matter which resists ID [13]. Each region is non-laminar with interspersed axon tracts and similar neuronal densities. In human and rodent, the estimated glucose use per neuron is remarkably constant, although it is at least 10x higher in the cerebral cortex than cerebellum [40]. All neurons depend on adjacent astrocytes to supply glucose (or lactate under metabolic stress) [41], so the high cortical consumption could reflect the high glia/neuron ratio in neocortex (3.72) vs. the cerebellum (0.23) [42] [40]. Regardless, both regions are highly susceptible to strong ID [43].

 A Na/K pump ATPase whose isoform constituents function better under ischemic conditions should reduce AD strength as well as support recovery. Our results suggest regional differences in the Na^+^/K^+^ pump strength during simulated ischemia because inhibiting the pump with ouabain mimics OGD`s effects which is distinct for each neuronal type. *It is important to note that not all Na* ^*+*^ */K* ^*+*^ *pump isoforms are completely inhibited by 100 µM ouabain or by OGD for 10 minutes*. Certain isoform combinations pump at higher rates during depolarization or ischemia [44][45] [46]. Variation in pump isoform type, density and or distribution can determine selective vulnerability to ischemia and so regional differences in pump isoforms merit further investigation [46]. Understanding how lower neurons naturally protect themselves from ischemia will help identify molecular targets in neocortex, hippocampus, striatum and thalamus to improve survival of these susceptible areas following stroke.

## Supporting Information

Table S1 **Whole-cell recording parameters from anteromedial ventral (AMV) thalamic neurons in response to 10 min OGD.** Newly acquired neurons could not be obtained post-OGD. Abbreviations: OGD Dur., oxygen/glucose deprivation duration; [Gluc.], glucose concentration; Rmp, resting membrane potential; Max Depol., maximum depolarization of anoxic depolarization; AP Ampl., action potential amplitude; Rin, whole-cell input resistance; Rin recovery of >115 % probably included partial micropipette block. These values were not included in the mean. % Rmp recovery was calculated before correcting for a +14 mV junction potential.
(DOCX)

Table S2 **Whole-cell recording parameters from additional midline thalamic neurons in response to OGD.** Eleven midline thalamic neurons from the lateral (n = 3), interoanteromedial (n =3), anteromedial (n = 1) and submedial thalamic nucleus (n = 1) were recorded during 10 minutes of OGD. Newly acquired neurons could not be obtained post-OGD. For abbreviations see Table S1.
(DOCX)

Table S3 **Whole-cell recording parameters from MNCs of PVN in response to OGD.** Eight MNCs were recorded during 10 minutes of OGD. Three newly acquired neurons were recorded post-OGD. For abbreviations see Table S1. *, AD onset values were obtained from PVN parvocellular imaging data.
(DOCX)

Table S4 **Whole-cell recording parameters from parvocellular neurons of PVN in response to OGD.** Twelve parvocellular neurons were recorded during 10 minutes of OGD. Eleven newly acquired neurons were recorded post-OGD. For abbreviations see Table S1. *, % Rin values could only be obtained for two neurons. % Rin recovery was 86% compared to post-OGD recordings (not shown).
(DOCX)

Table S5 **Whole-cell recording parameters from SCN neurons in response to OGD.** SCN neurons were recorded during 10 (n = 8) or 15 (n = 10) minutes of OGD. Fifteen newly acquired neurons were recorded post-OGD. For abbreviations see Table S1. *, increased % Rin values reflect broad Rin range of heterogeneous cell types in SCN.
(DOCX)
