# Supplementary material for: A Distinct Boundary between the Higher Brain’s Susceptibility to Ischemia and the Lower Brain’s Resistance
Source: PLoS One. 2013 Nov 6;8(11):e79589. doi: 10.1371/journal.pone.0079589 (PMC3819273; doi:10.1371/journal.pone.0079589)
Supplement: Table S1 — Whole-cell recording parameters from anteromedial ventral (AMV) thalamic neurons in response to 10 min OGD. Newly acquired neurons could not be obtained post-OGD. Abbreviations: OGD Dur., oxygen/glucose deprivation duration; [Gluc.], glucose concentration; Rmp, resting membrane potential; Max Depol., maximum depolarization of anoxic depolarization; AP Ampl., action potential amplitude; Rin, whole-cell input resistance; Rin recovery of >115 % probably included partial micropipette block. These values were not included in the mean. % Rmp recovery was calculated before correcting for a +14 mV junction potential. (DOCX) [file pone.0079589.s001.docx]

Supporting Table S1. Whole-cell recording parameters from anteromedial ventral (AMV) thalamic neurons in response to 10 min OGD.

| **OGD Dur. (min)** | **Rmp (mV)** | **Rmp Post-OGD (mV)** | **Max Depol. (mV)** | **AP Ampl. (mV)** | **Rin (MΩ)** | **AD Onset (s)** | **AD Rate (mV/s)** |
| --- | --- | --- | --- | --- | --- | --- | --- |
| 10 | -73 | lost | -2 | 74 | 66 | 256 | 1.44 |
| 10 | -66 | lost | -1 | 83 | 65 | 209 | 3.98 |
| 10 | -69 | lost | -2 | 85 | 117 | 216 | 2.3 |
| 10 | -67 | lost | -4 | 77 | 71 | 193 | 1.76 |
| 10 | -74 | lost | -2 | 82 | 64 | 190 | 2.57 |
| 10 | -70 | lost | -4 | 72 | 79 | 193 | 1.33 |
| 10 | -71 | lost | -2 | 83 | 73 | 244 | 1.72 |
| 10 | -74 | lost | -6 | 83 | 74 | 258 | 1.25 |
| 10 | -74 | lost | -7 | 70 | 81 | 189 | 2.65 |
| 10 | -69 | lost | -3 | 70 | 124 | 215 | 1.05 |
| 10 | -66 | lost | -9 | 83 | 81 | 220 | 1.98 |
| **MEAN** | -70 |  | -4 | 78 | 81 | 217 | 2.0 |
| **STDEV (±)** | 3.5 |  | 2.5 | 5.9 | 20 | 26 | 0.8 |

Newly acquired neurons could not be obtained post-OGD. Abbreviations: OGD Dur., oxygen/glucose deprivation duration; [Gluc.], glucose concentration; Rmp, resting membrane potential; Max Depol., maximum depolarization of anoxic depolarization; AP Ampl., action potential amplitude; Rin, whole-cell input resistance; Rin recovery of >115 % probably included partial micropipette block. These values were not included in the mean. % Rmp recovery was calculated before correcting for a +14 mV junction potential.
