# Supplementary material for: A Distinct Boundary between the Higher Brain’s Susceptibility to Ischemia and the Lower Brain’s Resistance
Source: PLoS One. 2013 Nov 6;8(11):e79589. doi: 10.1371/journal.pone.0079589 (PMC3819273; doi:10.1371/journal.pone.0079589)
Supplement: Table S2 — Whole-cell recording parameters from additional midline thalamic neurons in response to OGD. Eleven midline thalamic neurons from the lateral (n = 3), interoanteromedial (n =3), anteromedial (n = 1) and submedial thalamic nucleus (n = 1) were recorded during 10 minutes of OGD. Newly acquired neurons could not be obtained post-OGD. For abbreviations see Table S1. (DOCX) [file pone.0079589.s002.docx]

Supporting Table S2. Whole-cell recording parameters from additional midline thalamic neurons in response to OGD.

| **OGD Dur. (min)** | **Rmp (mV)** | **Rmp Post-OGD (mV)** | **Max Depol. (mV)** | **AP Ampl. (mV)** | **Rin (MΩ)** | **AD Onset (s)** | **AD Rate (mV/s)** |
| --- | --- | --- | --- | --- | --- | --- | --- |
| 10 | -70 | lost | -8 | 62 | 203 | 298 | 2.38 |
| 10 | -72 | lost | -3 | 66 | 45 | 210 | 9.8 |
| 10 | -72 | lost | -3 | 52 | 75 | 22 | 15.7 |
| 10 | -70 | lost | -4 | 70 | 100 | 257 | 8 |
| 10 | -70 | lost | -3 | 62 | 135 | 255 | 6.2 |
| 10 | -67 | lost | -2 | 45 | 208 | 242 | 2.8 |
| 10 | -64 | 0 | 0 | 59 | 222 | 216 | 1.2 |
| **MEAN** | -69 |  | -3.3 | 59 | 141 | 243 | 6.6 |
| **STDEV (±)** | 2.9 |  | 2.4 | 8.5 | 71 | 31 | 5.1 |

Eleven midline thalamic neurons from the lateral (n = 3), interoanteromedial (n =3), anteromedial (n = 1) and submedial thalamic nucleus (n = 1) were recorded during 10 minutes of OGD. Newly acquired neurons could not be obtained post-OGD. For abbreviations see Table 1.
