# Supplementary material for: A Distinct Boundary between the Higher Brain’s Susceptibility to Ischemia and the Lower Brain’s Resistance
Source: PLoS One. 2013 Nov 6;8(11):e79589. doi: 10.1371/journal.pone.0079589 (PMC3819273; doi:10.1371/journal.pone.0079589)
Supplement: Table S3 — Whole-cell recording parameters from MNCs of PVN in response to OGD. Eight MNCs were recorded during 10 minutes of OGD. Three newly acquired neurons were recorded post-OGD. For abbreviations see Table S1. *, AD onset values were obtained from PVN parvocellular imaging data. (DOCX) [file pone.0079589.s003.docx]

Supporting Table S3. Whole-cell recording parameters from MNCs of PVN in response to OGD.

| **OGD Dur. (min)** | **[Gluc.] (mM)** | **Rmp (mV)** | **Rmp Post-OGD (mV)** | **% Rmp Recov.** | **Max Depol. (mV)** | **AP Ampl. (mV)** | **AP Ampl. Post-OGD (mV)** | **% AP Ampl. Recov.** | **Rin (MΩ)** | **Rin Post-OGD (MΩ)** | **% Rin Recov.** | **AD Onset (s)** | **NEWLY ACQUIRED POST-OGD RECORDINGS** | | | | |
| --- | --- | --- | --- | --- | --- | --- | --- | --- | --- | --- | --- | --- | --- | --- | --- | --- | --- |
|  |  |  |  |  |  |  |  |  |  |  |  |  | **RMP (mV)** | **AP Ampl (mV)** | | | **Rin (MΩ)** |
| 10 | 0 | -51 | lost | N/A | -15 | 83 | N/A | N/A | 483 | N/A | N/A | - | -54 | | 88 | 666 | |
| 10 | 0 | -48 | -43 | 89.6 | -14 | 93 | 67 | 72 | 581 | 560 | 96 | - | -51 | | 83 | 780 | |
| 10 | 0 | -44 | -38 | 86.4 | -7 | 92 | 72 | 78 | 692 | 523 | 76 | - | -50 | | 93 | 577 | |
| 10 | 0 | -56 | -42 | 75.0 | -4 | 78 | 77 | 99 | 387 | 308 | 80 | - | - | | - | - | |
| 10 | 0 | -50 | lost | N/A | -17 | 85 | N/A | N/A | 834 | N/A | N/A | - | - | | - | - | |
| 10 | 0 | -55 | lost | N/A | 0 | 87 | N/A | N/A | 390 | N/A | N/A | - | - | | - | - | |
| 10 | 0 | -53 | -45 | 84.9 | -2 | 85 | 68 | 80 | 426 | 439 | 103 | - | - | | - | - | |
| 10 | 0 | -50 | lost | N/A | -12 | 84 | N/A | N/A | 340 | N/A | N/A | - | - | | - | - | |
| **MEAN** | | -51 | -42 | 84 | -9 | 86 | 71 | 82 | 517 | 458 | 89 | 298* | -52 | | 88 | 674 | |
| **STDEV (±)** | | 3.9 | 2.9 | 6.3 | 6.5 | 4.9 | 4.5 | 11.5 | 173 | 112 | 13 | 57 | 2.1 | | 5.0 | 102 | |

Eight MNCs were recorded during 10 minutes of OGD. Three newly acquired neurons were recorded post-OGD. For abbreviations see Table 1.

*, AD onset values were obtained from PVN parvocellular imaging data.
