# Supplementary material for: A Distinct Boundary between the Higher Brain’s Susceptibility to Ischemia and the Lower Brain’s Resistance
Source: PLoS One. 2013 Nov 6;8(11):e79589. doi: 10.1371/journal.pone.0079589 (PMC3819273; doi:10.1371/journal.pone.0079589)
Supplement: Table S4 — Whole-cell recording parameters from parvocellular neurons of PVN in response to OGD. Twelve parvocellular neurons were recorded during 10 minutes of OGD. Eleven newly acquired neurons were recorded post-OGD. For abbreviations see Table S1. *, % Rin values could only be obtained for two neurons. % Rin recovery was 86% compared to post-OGD recordings (not shown). (DOCX) [file pone.0079589.s004.docx]

Supporting Table S4. Whole-cell recording parameters from parvocellular neurons of PVN in response to OGD.

| **OGD Dur. (min)** | **[Gluc.] (mM)** | **Rmp (mV)** | **Rmp Post-OGD (mV)** | **% Rmp Recov.** | **Max Depol. (mV)** | **AP Ampl. (mV)** | **AP Ampl. Post-OGD (mV)** | **% AP Ampl. Recov.** | **Rin (MΩ)** | **Rin Post-OGD (MΩ)** | **% Rin Recov.** | **AD Onset (s)** | **NEWLY ACQUIRED POST-OGD RECORDINGS** | | | | |
| --- | --- | --- | --- | --- | --- | --- | --- | --- | --- | --- | --- | --- | --- | --- | --- | --- | --- |
|  |  |  |  |  |  |  |  |  |  |  |  |  | **RMP (mV)** | **AP Ampl (mV)** | | | **Rin (MΩ)** |
| 10 | 0 | -53 | Lost | N/A | -3 | 87 | N/A | N/A | 544 | N/A | N/A | 300 | -49 | | 83 | 675 | |
| 10 | 0 | -53 | lost | N/A | -2 | 75 | N/A | N/A | 944 | N/A | N/A | 312 | -47 | | 86 | 445 | |
| 10 | 0 | -44 | lost | N/A | -8 | 72 | N/A | N/A | 826 | N/A | N/A | 220 | -44 | | 89 | 513 | |
| 10 | 0 | -54 | -44 | 81 | -2 | 87 | 86 | 99 | 670 | 445 | 66 | 324 | -48 | | 78 | 440 | |
| 10 | 0 | -43 | -40 | 93 | -10 | 72 | N/A | N/A | 608 | N/A | N/A | 250 | -45 | | 71 | 900 | |
| 10 | 0 | -47 | lost | N/A | -8 | 72 | N/A | N/A | 677 | N/A | N/A | 383 | -44 | | 70 | 858 | |
| 10 | 0 | -52 | lost | N/A | -10 | 88 | N/A | N/A | 1158 | N/A | N/A | N/A | -43 | | 72 | 562 | |
| 10 | 0 | -51 | lost | N/A | -7 | 70 | N/A | N/A | 558 | N/A | N/A | N/A | -41 | | 89 | 544 | |
| 10 | 0 | -52 | -31 | 60 | -6 | 86 | 78 | 91 | 415 | 492 | 119 | N/A | -45 | | 88 | 615 | |
| 10 | 0 | -50 | lost | N/A | -4 | 75 | N/A | N/A | N/A | N/A | N/A | N/A | -31 | | 78 | 492 | |
| 10 | 0 | -45 | lost | N/A | -7 | 90 | N/A | N/A | 916 | N/A | N/A | N/A | -52 | | 73 | 592 | |
| 10 | 0 | -43 | lost | N/A | -1 | 89 | N/A | N/A | 426 | N/A | N/A | N/A | - | | - | - | |
| **MEAN** | | -49 | -38 | 78 | -6 | 80 | 82 | 95 | 704 | 469 | 92* | 298 | -44 | | 80 | 603 | |
| **STDEV (±)** | | 4.2 | 6.7 | 17.0 | 3.2 | 8.1 | 5.7 | 5.8 | 233 | 33 | 37 | 57 | 5.4 | | 7.6 | 154 | |

Twelve parvocellular neurons were recorded during 10 minutes of OGD. Eleven newly acquired neurons were recorded post-OGD. For abbreviations see Table 1.

*, % Rin values could only be obtained for two neurons. % Rin recovery was 86% compared to post-OGD recordings (not shown).
