# Supplementary material for: A Distinct Boundary between the Higher Brain’s Susceptibility to Ischemia and the Lower Brain’s Resistance
Source: PLoS One. 2013 Nov 6;8(11):e79589. doi: 10.1371/journal.pone.0079589 (PMC3819273; doi:10.1371/journal.pone.0079589)
Supplement: Table S5 — Whole-cell recording parameters from SCN neurons in response to OGD. SCN neurons were recorded during 10 (n = 8) or 15 (n = 10) minutes of OGD. Fifteen newly acquired neurons were recorded post-OGD. For abbreviations see Table S1. *, increased % Rin values reflect broad Rin range of heterogeneous cell types in SCN. (DOCX) [file pone.0079589.s005.docx]

Supporting Table S5. Whole-cell recording parameters from SCN neurons in response to OGD.

| **OGD Dur. (min)** | **[Gluc.] (mM)** | **Rmp (mV)** | **Rmp Post-OGD (mV)** | **% Rmp Recov.** | **Max Depol. (mV)** | **AP Ampl. (mV)** | **AP Ampl. Post-OGD (mV)** | **% AP Ampl. Recov.** | **Rin (MΩ)** | **Rin Post-OGD (MΩ)** | **% Rin Recov.** | **AD Onset (s)** | **NEWLY ACQUIRED POST-OGD RECORDINGS** | | | | |
| --- | --- | --- | --- | --- | --- | --- | --- | --- | --- | --- | --- | --- | --- | --- | --- | --- | --- |
|  |  |  |  |  |  |  |  |  |  |  |  |  | **RMP (mV)** | | **AP Ampl (mV)** | | **Rin (MΩ)** |
| 15 | 0 | -47 | -55 | 87 | -17 | 71 | N/A | N/A | 1017 | 969 | 95 | 448 | -61 | 76 | | 1254 | |
| 15 | 0 | -40 | lost | N/A | -25 | 69 | N/A | N/A | 414 | lost | N/A | 445 | -59 | 72 | | 359 | |
| 15 | 0 | -46 | -52 | 83 | -2 | 75 | N/A | N/A | 686 | N/A | N/A | N/A | -56 | 66 | | 367 | |
| 15 | 0 | -49 | -52 | 78 | -13 | 87 | 43 | 49 | 850 | 900 | 106 | N/A | -59 | 77 | | 756 | |
| 15 | 0 | -40 | -43 | 73 | -20 | 80 | N/A | N/A | 747 | 664 | 89 | 422 | -51 | 66 | | 2300 | |
| 15 | 0 | -50 | lost | N/A | -25 | 77 | 33 | 43 | 400 | N/A | lost | N/A | -59 | 63 | | 916 | |
| 15 | 0 | -43 | -55 | 95 | -25 | 72 | N/A | N/A | 703 | N/A | N/A | 360 | -56 | 59 | | 658 | |
| 15 | 0 | -50 | -55 | 82 | -8 | 80 | N/A | N/A | 676 | N/A | N/A | 500 | -60 | 83 | | 791 | |
| 15 | 0 | -50 | -64 | 100 | -23 | 72 | 49 | 68 | 851 | 1063 | >115 | 503 | -53 | 56 | | 1229 | |
| 15 | 0 | -41 | lost | N/A | -25 | 88 | N/A | N/A | 912 | lost | N/A | N/A | -50 | 56 | | 1347 | |
| 10 | 0 | -50 | -47 | 66 | -13 | 76 | N/A | N/A | 872 | N/A | N/A | N/A | -59 | 74 | | 1185 | |
| 10 | 0 | -45 | -26 | 27 | lost | 78 | N/A | N/A | 824 | N/A | N/A | N/A | -59 | 81 | | 748 | |
| 10 | 0 | -44 | -40 | 59 | -25 | 99 | N/A | N/A | 873 | N/A | N/A | 340 | -58 | 72 | | 319 | |
| 10 | 0 | -39 | -55 | 100 | -24 | 68 | 61 | 90 | 395 | 451 | 114 | N/A | -58 | 79 | | 945 | |
| 10 | 0 | -44 | -53 | 89 | -16 | 56 | 34 | 61 | 608 | 678 | 112 | N/A | -57 | 78 | | 325 | |
| 10 | 0 | -52 | -70 | 100 | -19 | 72 | 57 | 79 | 1499 | 1261 | 84 | N/A | - | - | | - | |
| 10 | 0 | -42 | lost | N/A | -21 | 67 | N/A | N/A | 1370 | lost | N/A | N/A | - | - | | - | |
| 10 | 0 | -43 | lost | N/A | -25 | 85 | N/A | N/A | 676 | lost | N/A | N/A | - | - | | - | |
| **MEAN** | | -45 | -37 | 81 | -19 | 76 | 46 | 65 | 799 | 855 | 100* | 431 | -57 | 71 | | 900 | |
| **STDEV (±)** | | 4.1 | 10.9 | 21.8 | 6.9 | 10 | 12 | 18 | 293 | 275 | 15 | 63 | 3.3 | 9.0 | | 523 | |

SCN neurons were recorded during 10 (n = 8) or 15 (n = 10) minutes of OGD. Fifteen newly acquired neurons were recorded post-OGD. For abbreviations see Table 1. *, increased % Rin values reflect broad Rin range of heterogeneous cell types in SCN.
